# Supplementary material for: Phytochemicals and Their Correlation with Molecular Data in Micromeria and Clinopodium (Lamiaceae) Taxa
Source: Plants (Basel). 2022 Dec 6;11(23):3407. doi: 10.3390/plants11233407 (PMC9739532; doi:10.3390/plants11233407)
Supplement: Supplementary file 1 [file plants-11-03407-s001.zip › Table S1.pdf]

plants

Table S1. Phytochemical composition, identification and major groups of chemical components (%) of essential oil (EO) of *Micromeria cristata* ssp. *cristata* (Mc), and *M. cristata* ssp. *kosaninii* (McK).

			Sample and yield					
			Mc1	Mc2	Mc3	Mc4	Mc5	McK
			0.96	0.87	0.9	1.02	1.06	0.86
Component	RI[a]	RI[b]	EO±SD	EO±SD	EO±SD	EO±SD	EO±SD	EO±SD
**MH**			**12**	**10.67**	**10.99**	**8.14**	**5.77**	**1.13**
α-Pinene[*]	938	1025	0.20±0.02	0.67±0.01	0.58±0.01	0.50±0.01	-	-
Verbenene	960	1121	3.52±0.01	3.22±0.01	4.52±0.01	4.36±0.01	0.61±0.01	-
Camphene[*]	962	1056	1.75±0.01	0.79±0.02	0.52±0.02	0.64±0.02	3.91±0.01	-
Sabinene	971	1126	-	-	-	-	0.52±0.02	0.32±0.01
β-Pinene	982	1092	1.12±0.01	0.53±0.01	0.32±0.01	0.44±0.01	-	0.55±0.03
Myrcene[*]	992	1173	0.32±0.02	-	-	0.46±0.01	0.41±0.01	-
δ-3-Carene	1008	1147	-	-	-	-	0.32±0.01	-
α-Terpinene	1016	1192	0.87±0.01	-	-	-	-	-
β-Phellandrene	1025	1208	0.92±0.01	0.26±0.01	-	-	-	-
Limonene	1032	1204	0.43±0.01	0.75±0.01	1.35±0.01	-	-	-
γ-Terpinene	1057	1225	1.22±0.05	2.24±0.02	1.93±0.01	0.87±0.01	-	0.26±0.01
cis-Sabinene hydrate	1065	1456	1.43±0.01	2.21±0.01	1.54±0.01	0.73±0.01	-	-
allo-Ocimene	1128	1370	0.22±0.01	-	0.23±0.02	0.14±0.01	-	-
**OM**			**36.98**	**38.25**	**42.94**	**40.76**	**39.33**	**28.97**

trans -Linalool oxide *	1088	1434	0.12±0.01	0.32±0.02	0.41±0.01	-	0.53±0.01	-
Linalool*	1099	1548	0.82±0.01	0.52±0.01	-	-	-	1.32±0.01
cis-p-Menth-*2-en-1*-ol	1118	1600	-	-	0.31±0.01	-	-	3.95±0.01
β-Thujone	1121	1438	0.23±0.02	-	-	-	-	-
α-Campholenal	1129	1496	-	10.11±0.01	0.64±0.01	-	-	-
Isopulegol	1145	1564	-	-	-	-	-	0.83±0.01
trans-Pinocarveol	1147	1658	-	-	0.46±0.01	-	-	-
Menthone	1148	1462	-	-	-	-	-	0.44±0.03
Camphor*	1151	1499	2.17±0.01	4.34±0.01	3.25±0.01	0.22±0.01	0.61±0.01	-
Isoborneol	1155	1649	4.56±0.01	-	-	2.38±0.01	3.32±0.01	1.14±0.01
Pinocarvone	1160	1565	-	-	-	-	-	3.15±0.01
Borneol*	1176	1699	14.75±0.02	14.11±0.01	26.28±0.01	25.22±0.01	23.30±0.01	2.15±0.01
Terpinen-4-*ol*	1184	1601	2.31±0.01	1.61±0.05	0.73±0.01	0.42±0.01	0.39±0.01	1.65±0.01
α-Terpineol	1186	1686	0.22±0.01	0.53±0.01	0.44±0.01	-	0.25±0.03	0.54±0.01
Myrtenol	1197	1782	-	-	-	-	-	2.81±0.01
Verbenone	1204	1705	3.23±0.01	2.23±0.01	3.31±0.01	4.13±0.01	4.53±0.01	-
trans-Carveol	1215	1815	1.12±0.01	2.25±0.01	1.93±0.01	0.68±0.01	0.52±0.01	-
β-Cyclocitral	1223	1629	0.12±0.01	-	-	-	-	-
Pulegone	1233	1641	-	-	-	-	-	8.91±0.01
Piperitone	1250	1719	-	0.36±0.01	0.88±0.03	-	-	0.33±0.01
Linalyl acetate	1252	1553	0.11±0.01	0.65±0.03	-	1.66±0.01	0.62±0.01	0.82±0.01

Bornyl acetate	1285	1570	7.22±0.01	1.22±0.02	4.30±0.03	5.23±0.01	4.82±0.02	-
α-Terpenyl acetate	1349	1685	-	-	-	0.82±0.02	0.44±0.01	0.93±0.01
**SH**			**15.27**	**13.18**	**8.48**	**11.91**	**11.25**	**13.24**
α-Copaene	1377	1484	0.26±0.01	0.32±0.01	0.44±0.01	-	-	0.15±0.01
β-Bourbonene	1383	1508	1.15±0.01	0.32±0.01	0.21±0.01	0.25±0.01	0.25±0.01	0.35±0.03
β-Elemene	1389	1593	-	-	-	-	0.32±0.01	-
α-Gurjunene	1407	1520	0.16±0.02	0.93±0.01	0.15±0.01	-	-	0.91±0.01
E-Caryophyllene*	1424	1585	2.21±0.01	2.91±0.01	2.43±0.02	1.25±0.01	1.42±0.01	2.94±0.01
β-Copaene	1429	1584	0.41±0.01	1.65±0.01	0.43±0.01	0.45±0.01	0.21±0.01	1.15±0.01
trans-*α*-Bergamotene	1433	1580	0.61±0.01	0.28±0.01	0.33±0.01	1.26±0.01	1.10±0.01	0.23±0.01
(*Z*)-*β*-Farnesene	1454	1639	0.45±0.01	0.38±0.01	0.42±0.01	-	-	0.38±0.01
α-Humulene	1456	1654	0.16±0.01	0.26±0.01	0.13±0.01	1.25±0.01	1.31±0.01	-
allo-Aromadendrene*	1465	1662	1.17±0.03	1.34±0.03	0.72±0.01	-	-	1.91±0.01
β-Chamigrene	1477	1735	0.22±0.01	0.24±0.01	0.28±0.01	-	-	0.35±0.01
Germacrene D*	1481	1692	2.66±0.01	2.15±0.02	1.98±0.01	6.25±0.01	4.95±0.01	2.13±0.01
β-Bisabolene	1494	1729	1.22±0.02	0.21±0.03	0.43±0.01	-	0.31±0.01	0.21±0.01
Viridiflorene	1496	1697	1.54±0.01	1.26±0.01	-	-	-	1.18±0.01
Bicyclogermacrene	1500	1718	2.22±0.01	0.51±0.01	0.53±0.02	0.25±0.01	0.12±0.02	0.92±0.01
δ-Cadinene	1517	1745	0.83±0.01	0.42±0.01	-	0.95±0.01	1.26±0.01	0.43±0.01
**OS**			**23.23**	**23.61**	**26.02**	**23.86**	**27.72**	**34.82**
Spathulenol*	1577	2101	0.22±0.01	1.42±0.03	0.72±0.01	-	-	2.46±0.01

Caryophyllene oxide*	1581	1955	3.52±0.01	6.44±0.01	6.54±0.01	10.02±0.01	12.32±0.01	13.41±0.01
γ-Eudesmol	1632	2135	0.84±0.01	0.36±0.01	0.28±0.01	1.36±0.01	1.11±0.01	0.32±0.01
α-Muurolol	1645	2163	-	-	-	-	-	17.53±0.01
α-Cadinol	1655	2208	17.72±0.01	14.51±0.01	16.32±0.01	12.48±0.01	13.82±0.01	0.46±0.01
α-Bisabolol*	1688	2116	0.32±0.01	0.52±0.05	1.73±0.05	-	0.24±0.01	0.51±0.01
α-Bisabolol oxide	1748	2511	0.61±0.01	0.36±0.01	0.43±0.05	-	0.23±0.05	0.13±0.01
**PC**			**0.32**	**1.58**	**1.13**	**0.86**	**0.95**	**1.23**
Thymol*	1290	2198	0.32±0.01	1.58±0.01	1.13±0.01	0.86±0.01	0.95±0.01	1.23±0.02
**CC**			**0.91**	**1.97**	**1.54**	**1.70**	**1.12**	**1.34**
1-Octen-3-*ol*	974	1433	0.11±0.02	0.72±0.01	0.75±0.01	0.95±0.01	0.20±0.02	0.82±0.05
3-Octanol acetate	1125	1376	0.41±0.03	0.21±0.02	-	-	-	-
Isoamyl hexanoate	1256	1457	0.26±0.01	0.42±0.01	0.53±0.01	-	-	-
β-Ionone	1487	1924	0.13±0.01	0.62±0.01	0.26±0.01	0.75±0.01	0.92±0.01	0.52±0.01
**H**			**3.14**	**3.58**	**3.09**	**3.30**	**4.86**	**11.50**
Eicosane*	2000	2000	-	-	0.42±0.01	0.65±0.01	0.81±0.01	-
Heneicosane*	2100	2100	-	0.24±0.01	0.88±0.01	0.95±0.01	1.12±0.01	-
Docosane*	2200	2200	2.31±0.03	1.61±0.01	0.46±0.01	1.25±0.01	2.61±0.01	5.72±0.01
Tricosane*	2300	2300	0.18±0.01	0.42±0.01	0.71±0.01	0.45±0.01	0.32±0.01	0.42±0.01
Pentacosane*	2500	2500	0.22±0.05	0.33±0.01	-	-	-	0.27±0.01
Hexacosane*	2600	2600	0.12±0.03	-	-	-	-	4.52±0.03
Heptacosane*	2700	2700	0.31±0.02	0.26±0.01	0.62±0.02	-	-	0.33±0.01

Octacosane*	2800	2800	-	0.72±0.02	-	-	-	0.24±0.05
Total identified (%)			**91.85**	**92.84**	**94.19**	**90.53**	**91.00**	**92.23**

Retention indices were determined relative to a series of *n*-alkanes (C_8–C_{40}) on capillary columns VF5-ms (RI^a) and CP Wax 52 (RI^b); identification method: RI comparison of RIs with those listed in a homemade library; reported in the literature [87] and/or authentic samples; comparison of mass spectra with those in mass spectral libraries NIST02 [88] and Wiley 9; *, injection reference compounds; SD, standard deviation; MH, Monoterpene hydrocarbons; OM, Oxygenated monoterpenes; SH, Sesquiterpene hydrocarbons; OS, Oxygenated sesquiterpenes; PC, Phenolic compounds; CC, Carbonylic compounds; H, Hydrocarbons.
