# Supplementary material for: Phytochemicals and Their Correlation with Molecular Data in Micromeria and Clinopodium (Lamiaceae) Taxa
Source: Plants (Basel). 2022 Dec 6;11(23):3407. doi: 10.3390/plants11233407 (PMC9739532; doi:10.3390/plants11233407)
Supplement: Supplementary file 1 [file plants-11-03407-s001.zip › Table S2.pdf]

plants MDPI

Table S2. Phytochemical composition, identification and major groups of chemical components (%) of essential oil (EO) of *Micromeria croatica* (Mcr).

			Sample and yield									
			Mcr1	Mcr2	Mcr3	Mcr4	Mcr5	Mcr6	Mcr7	Mcr8	Mcr9	Mcr10
			1.91	1.02	1.12	0.98	0.95	1.65	1.13	0.98	1.06	1.79
Component	RI[a]	RI[b]	EO±SD	EO±SD	EO±SD	EO±SD	EO±SD	EO±SD	EO±SD	EO±SD	EO±SD	EO±SD
**MH**			**9.28**	**11.31**	**10.02**	**9.55**	**7.03**	**7.55**	**6.91**	**10.64**	**8.32**	**9.16**
α-Pinene*	938	1025	3.61±0.02	2.16±0.01	1.63±0.01	2.57±0.01	1.12±0.05	1.68±0.01	1.32±0.01	1.97±0.01	1.27±0.01	2.13±0.01
Verbenene	960	1121	0.31±0.01	0.84±0.01	0.77±0.05	0.76±0.01	0.22±0.01	0.48±0.03	0.45±0.01	0.59±0.01	0.52±0.01	0.22±0.01
Camphene*	962	1056	0.45±0.01	0.89±0.01	0.61±0.01	0.49±0.01	0.21±0.02	-	-	1.08±0.01	0.68±0.01	0.21±0.02
β-Pinene	982	1092	4.61±0.01	2.28±0.07	3.96±0.01	3.85±0.01	3.21±0.01	3.88±0.01	4.76±0.01	3.43±0.01	2.51±0.01	3.21±0.01
Myrcene*	992	1173	0.18±0.01	0.25±0.01	0.76±0.01	-	-	-	-	-	-	-
α-Terpinene	1016	1192	-	0.52±0.02	-	0.21±0.01	0.60±0.01	-	-	0.98±0.03	1.56±0.01	0.76±0.01

p-Cymene	1021	1270	-	0.92±0.01	0.97±0.01	-	0.22±0.01	0.42±0.01	.	1.05±0.01	0.65±0.03	0.82±0.01
β-Phellandrene	1025	1208	-	1.63±0.04	-	0.71±0.01	1.13±0.01	0.73±0.01	-	-	-	1.36±0.01
Limonene	1032	1204	0.12±0.03	1.82±0.01	1.32±0.01	0.96±0.01	0.32±0.01	0.36±0.01	0.38±0.01	1.54±0.01	1.13±0.01	0.45±0.05
**OM**			**38.90**	**33.99**	**37.65**	**41.89**	**30.07**	**31.86**	**37.56**	**37.76**	**38.64**	**36.58**
trans-Linalool oxide*	1088	1434	-	0.99±0.01	0.87±0.01	0.43±0.05	0.72±0.01	0.67±0.03	0.67±0.01	1.02±0.01	0.68±0.01	0.88±0.01
Linalool*	1099	1548	1.13±0.01	1.73±0.03	1.66±0.01	4.13±0.01	0.32±0.01	0.86±0.01	1.03±0.02	4.73±0.01	2.48±0.01	0.98±0.01
Isopulegol	1145	1564	-	-	-	-	0.52±0.02	0.62±0.01	-	-	-	1.05±0.01
trans-Pinocarveol	1147	1658	0.64±0.02	0.52±0.01	0.81±0.03	0.38±0.07	0.32±0.01	0.33±0.01	0.63±0.01	0.21±0.01	0.98±0.01	0.74±0.01
Camphor	1151	1499	6.34±0.01	2.31±0.01	5.44±0.01	5.15±0.01	4.82±0.01	3.34±0.01	5.24±0.01	1.29±0.01	5.29±0.01	3.89±0.01
Pinocarvone	1160	1565	1.35±0.02	1.33±0.01	1.86±0.01	2.02±0.01	2.26±0.01	2.24±0.01	1.84±0.01	1.49±0.01	1.95±0.01	2.75±0.01
Borneol*	1176	1699	17.26±0.01	19.45±0.01	18.77±0.01	23.43±0.01	16.13±0.02	18.36±0.01	18.13±0.01	21.43±0.01	16.48±0.01	18.44±0.01
Terpinen-4-*ol*	1184	1601	0.62±0.05	-	-	-	-	0.58±0.02	-	-	-	-
Myrtenol	1197	1782	-	-	0.82±0.01	-	0.42±0.01	0.48±0.01	0.53±0.05	0.36±0.01	0.76±0.01	0.79±0.01

Verbenone	1204	1705	-	0.76±0.01	0.98±0.03	-	0.33±0.05	0.39±0.01	0.68±0.01	-	0.73±0.05	-
trans-Carveol	1215	1815	0.42±0.01	0.39±0.07	-	0.62±0.01	0.32±0.01	0.87±0.02	0.94±0.01	-	0.37±0.01	0.66±0.01
endo-Fenchyl acetate	1218	1465	7.62±0.01	3.75±0.01	3.68±0.01	3.32±0.01	2.83±0.01	2.49±0.01	6.44±0.01	4.06±0.01	6.15±0.01	4.86±0.01
Piperitone	1250	1719	2.12±0.02	-	0.76±0.01	-	-	-	0.49±0.01	0.82±0.05	0.31±0.01	-
Bornyl acetate	1285	1570	0.63±0.05	0.72±0.01	1.02±0.01	1.65±0.01	-	-	-	0.86±0.01	-	-
α-Terpenyl acetate	1349	1685	-	0.88±0.01	-	-	0.33±0.01	-	0.59±0.03	0.38±0.01	0.98±0.01	0.83±0.01
Neryl acetate	1358	1692	0.54±0.01	0.29±0.01	0.42±0.01	0.76±0.01	0.64±0.01	-	-	0.77±0.02	0.77±0.02	-
**SH**			**31.38**	**29.15**	**25.51**	**26.09**	**30.19**	**35.73**	**31.40**	**30.33**	**30.75**	**31.75**
α-Copaene	1377	1484	0.20±0.05	0.98±0.02	1.87±0.01	2.15±0.01	0.74±0.03	0.68±0.01	0.49±0.01	1.95±0.01	0.24±0.01	0.24±0.01
β-Bourbonene	1383	1508	2.31±0.01	2.09±0.01	2.45±0.01	2.56±0.01	1.12±0.02	3.73±0.01	2.98±0.01	2.78±0.01	3.46±0.01	3.96±0.01
β-Elemene	1389	1593	1.15±0.01	-	-	-	-	1.61±0.01	-	-	-	-
E-Caryophyllene*	1424	1585	10.60±0.01	12.45±0.01	12.87±0.01	12.07±0.01	11.13±0.01	10.95±0.01	9.95±0.01	14.33±0.01	7.66±0.01	7.13±0.01
β-Copaene	1429	1584	0.73±0.03	0.67±0.01	0.89±0.01	0.11±0.01	0.50±0.01	0.43±0.03	0.48±0.01	0.21±0.01	0.63±0.03	0.58±0.01

trans-α-Bergamotene	1433	1580	0.14±0.01	0.87±0.05	0.24±0.01	1.67±0.01	-	-	-	0.38±0.01	-	-
α-Humulene	1456	1654	1.72±0.01	0.53±0.01	-	0.63±0.01	1.28±0.01	2.16±0.01	1.97±0.01	-	1.78±0.01	2.68±0.01
allo-Aromadendrene[*]	1465	1662	1.21±0.01	-	0.50±0.01	-	-	1.72±0.01	0.88±0.01	0.78±0.05	2.03±0.01	1.32±0.01
β-Chamigrene	1477	1735	-	-	-	-	0.32±0.01	-	-	-	-	-
Germacrene D[*]	1481	1692	12.10±0.02	10.78±0.01	6.26±0.01	6.25±0.01	13.12±0.01	13.66±0.01	13.86±0.01	9.03±0.01	8.62±0.01	14.12±0.01
β-Bisabolene	1494	1729	1.22±0.01	0.78±0.01	0.43±0.03	0.65±0.07	0.73±0.01	0.79±0.01	0.79±0.01	0.87±0.07	2.58±0.01	0.79±0.03
Viridiflorene	1496	1697	-	-	-	-	0.82±0.01	-	-	-	-	-
β-Curcumene	1514	1731	-	-	-	-	-	-	-	-	2.12±0.01	-
δ-Cadinene	1517	1745	-	-	-	-	0.43±0.02	-	-	-	-	0.93±0.01
**OS**			**12.79**	**16.40**	**15.68**	**13.38**	**18.89**	**17.61**	**16.32**	**13.23**	**13.89**	**14.34**
Spathulenol[*]	1577	2101	0.32±0.01	-	-	-	-	-	-	-	-	-
Caryophyllene oxide[*]	1581	1955	10.92±0.01	14.05±0.01	12.44±0.01	12.07±0.01	15.75±0.01	15.44±0.01	14.64±0.01	12.65±0.01	12.34±0.01	13.15±0.01
γ-Eudesmol	1632	2135	0.91±0.01	0.76±0.01	0.52±0.01	0.32±0.03	0.66±0.01	0.75±0.01	0.75±0.01	0.14±0.01	0.39±0.01	0.35±0.05

α-Cadinol	1655	2208	0.21±0.01	0.83±0.01	-	0.53±0.01	-	-	-	-	-	-
α-Bisabolol*	1688	2116	0.43±0.03	0.76±0.01	2.72±0.03	0.46±0.01	2.13±0.01	1.21±0.01	0.93±0.01	0.44±0.01	1.16±0.01	0.56±0.01
α-Bisabolol oxide	1748	2511	-	-	-	-	0.35±0.01	0.21±0.02	-	-	-	0.28±0.01
**PC**			**0.33**	**-**	**-**	**-**	-	**-**	**0.49**	**-**	**0.47**	**0.68**
Eugenol*	1370	2175	0.33±0.01	-	-	-	-	-	0.49±0.01	-	0.47±0.05	0.68±0.03
**CC**			**0.61**	**-**	**0.89**	**0.48**	**1.89**	**0.72**	**1.61**	**-**	**1.65**	**2.56**
1-Octen-3-*ol*	974	1433	0.51±0.01	-	0.46±0.01	0.48±0.01	0.42±0.01	-	0.69±0.01	-	0.67±0.01	0.48±0.01
Isobutyl hexanoate	1155	1370	-	-	0.43±0.03	-	1.32±0.01	0.72±0.01	0.92±0.05	-	0.98±0.01	1.32±0.01
β-Ionone	1487	1924	0.10±0.01	-	-	-	0.15±0.01	-	-	-	-	0.76±0.01
**H**			**1.07**	**0.25**	**1.27**	**0.77**	**2.72**	**3.12**	**3.08**	**0.86**	**2.45**	**2.84**
Eicosane*	2000	2000	0.31±0.02	-	0.13±0.01	-	0.42±0.01	0.58±0.01	0.33±0.01	0.17±0.01	0.43±0.01	0.85±0.03
Docosane*	2200	2200	-	-	0.80±0.05	-	2.12±0.03	1.86±0.01	1.93±0.01	0.69±0.01	1.65±0.01	1.72±0.01
Pentacosane*	2500	2500	0.31±0.01	0.25±0.01	0.34±0.01	0.34±0.01	0.18±0.05	0.68±0.05	0.34±0.01	-	0.37±0.01	0.27±0.01

Heptacosane*	2700	2700	0.33±0.02	-	-	0.43±0.01	-	-	0.48±0.01	-	-	-
***Total identified* (%)**			**94.36**	**91.1**	**91.02**	**92.16**	**90.79**	**96.59**	**97.37**	**92.82**	**96.17**	**97.91**

Retention indices were determined relative to a series of *n*-alkanes (C_8–C_{40}) on capillary columns VF5-ms (RI^a) and CP Wax 52 (RI^b); identification method: RI comparison of RIs with those listed in a homemade library; reported in the literature [87] and/or authentic samples; comparison of mass spectra with those in mass spectral libraries NIST02 [88] and Wiley 9; *, injection reference compounds; SD, standard deviation; MH, Monoterpene hydrocarbons; OM, Oxygenated monoterpenes; SH, Sesquiterpene hydrocarbons; OS, Oxygenated sesquiterpenes; PC, Phenolic compounds; CC, Carbonylic compounds; H, Hydrocarbons.
