# Supplementary material for: Phytochemicals and Their Correlation with Molecular Data in Micromeria and Clinopodium (Lamiaceae) Taxa
Source: Plants (Basel). 2022 Dec 6;11(23):3407. doi: 10.3390/plants11233407 (PMC9739532; doi:10.3390/plants11233407)
Supplement: Supplementary file 1 [file plants-11-03407-s001.zip › Table S3.pdf]

plants

**Table S3.** Phytochemical composition, identification and major groups of chemical components (%) of essential oil (EO) of *Micromeria croatica* (McrP), *M. graeca* ssp. *graeca* (Mg), and *M. graeca* ssp. *fruticulosa* (MgF).

| | | | Sample and yield | | | | |
|---|---|---|---|---|---|---|---|
| | | | McrP1 | McrP2 | Mg1 | Mg2 | MgF |
| | | | 1.05 | 0.95 | 1.18 | 1.22 | 0.98 |
| Component | RI[a] | RI[b] | EO±SD | EO±SD | EO±SD | EO±SD | EO±SD |
| **MH** | | | **1.66** | **4.26** | **5.70** | **4.96** | **8.43** |
| *α*-Thujene | 924 | 1029 | 0.25±0.01 | - | 0.58±0.02 | 0.28±0.01 | - |
| *α*-Pinene* | 938 | 1025 | 0.54±0.03 | - | 1.72±0.01 | 0.92±0.01 | 2.23±0.01 |
| Camphene* | 962 | 1056 | 0.56±0.01 | 3.21±0.03 | - | 0.53±0.01 | 0.92±0.01 |
| *β*-Pinene | 982 | 1092 | - | - | 2.13±0.01 | 2.21±0.02 | 0.63±0.01 |
| Myrcene | 992 | 1173 | 0.31±0.01 | - | - | - | 0.49±0.01 |
| *α*-Terpinene | 1016 | 1192 | - | - | - | - | 0.68±0.01 |
| *p*-Cymene | 1021 | 1270 | - | 0.51±0.01 | - | - | 1.69±0.01 |
| *β*-Phellandrene | 1025 | 1208 | - | 0.54±0.03 | - | - | 0.95±0.05 |
| Limonene | 1032 | 1204 | - | - | 0.86±0.01 | 0.61±0.01 | 0.67±0.01 |
| *(Z)*-*β*-Ocimene* | 1052 | 1218 | - | - | 0.41±0.01 | 0.41±0.01 | - |
| *allo*-Ocimene | 1128 | 1370 | - | - | - | - | 0.17±0.03 |
| **OM** | | | **57.76** | **54.59** | **20.58** | **19.05** | **37.36** |
| *trans*-Linalool oxide * | 1088 | 1434 | - | 0.41±0.01 | 7.28±0.01 | 5.32±0.01 | - |
| Linalool* | 1099 | 1548 | - | - | 3.97±0.01 | 5.67±0.01 | 4.37±0.01 |

| | | | | | | | |
|---|---|---|---|---|---|---|---|
| β-Thujone | 1121 | 1438 | - | - | - | 0.86±0.01 | - |
| *α*-Campholenal | 1129 | 1496 | 0.34±0.01 | - | - | - | - |
| *trans*-Pinocarveol | 1147 | 1658 | - | 0.66±0.01 | 2.33±0.01 | 2.15±0.01 | 0.93±0.01 |
| Camphor | 1151 | 1499 | 16.50±0.01 | 13.80±0.01 | 0.62±0.03 | 1.03±0.02 | 1.29±0.01 |
| Pinocarvone | 1160 | 1565 | - | - | - | - | 17.39±0.01 |
| Borneol* | 1176 | 1699 | 26.46±0.01 | 28.71±0.01 | 1.40±0.03 | 0.27±0.01 | 8.29±0.01 |
| Terpinen-4-*ol* | 1184 | 1601 | 0.32±0.01 | - | 0.55±0.01 | 0.59±0.01 | - |
| *α*-Terpineol | 1186 | 1686 | 0.25±0.01 | - | - | - | 0.62±0.05 |
| Myrtenol | 1197 | 1782 | - | - | - | - | 0.48±0.02 |
| Verbenone | 1204 | 1705 | - | 2.13±0.01 | 0.63±0.01 | 0.77±0.01 | 0.39±0.01 |
| *trans*-Carveol | 1215 | 1815 | 0.54±0.01 | 0.45±0.01 | 0.43±0.01 | 0.87±0.01 | 0.88±0.01 |
| *endo*-Fenchyl acetate | 1218 | 1465 | - | - | 2.13±0.01 | 1.52±0.01 | 0.92±0.01 |
| *β*-Cyclocitral | 1223 | 1629 | - | - | - | - | 0.72±0.03 |
| Pulegone | 1234 | 1641 | - | 0.84±0.01 | - | - | - |
| Linalyl acetate | 1252 | 1553 | - | - | 0.66±0.05 | - | 0.26±0.01 |
| Bornyl acetate | 1285 | 1570 | 1.56±0.01 | 1.25±0.01 | 0.58±0.02 | - | - |
| Piperitonene | 1340 | 1882 | 9.53±0.01 | 4.51±0.01 | - | - | - |
| Neryl acetate | 1358 | 1692 | - | 0.52±0.01 | - | - | 0.82±0.05 |
| Piperitenone oxide | 1366 | 1941 | 2.26±0.01 | 1.31±0.01 | - | - | - |
| **SH** | | | **23.26** | **27.80** | **20.81** | **20.76** | **17.74** |
| *α*-Copaene | 1377 | 1484 | - | 0.25±0.01 | 0.75±0.07 | 0.77±0.01 | 2.45±0.01 |

| | | | | | | | |
|---|---|---|---|---|---|---|---|
| *β*-Bourbonene | 1383 | 1508 | - | 0.42±0.01 | 0.77±0.01 | 0.51±0.03 | 0.83±0.01 |
| *β*-Elemene | 1389 | 1593 | 0.82±0.01 | - | - | - | 1.77±0.01 |
| *α*-Gurjunene | 1407 | 1520 | - | - | 1.36±0.01 | 0.58±0.01 | 0.51±0.01 |
| *E*-Caryophyllene* | 1424 | 1585 | 11.4±0.01 | 16.8±0.01 | 0.32±0.01 | 0.67±0.03 | - |
| *β*-Copaene | 1429 | 1584 | - | 0.71±0.01 | 2.68±0.01 | 4.82±0.01 | - |
| *trans*-*α*-Bergamotene | 1433 | 1580 | - | 0.15±0.01 | - | - | 3.58±0.01 |
| (Z)-*β*-Farnesene | 1454 | 1639 | - | - | - | - | 0.58±0.01 |
| *α*-Humulene | 1456 | 1654 | - | - | 1.45±0.01 | 1.26±0.01 | 0.71±0.01 |
| *allo*-Aromadendrene* | 1465 | 1662 | 1.44±0.01 | 1.24±0.01 | 7.38±0.01 | 6.82±0.01 | - |
| *β*-Chamigrene | 1477 | 1735 | - | - | 0.56±0.03 | 0.59±0.01 | 0.92±0.01 |
| Germacrene D | 1481 | 1692 | 2.95±0.01 | 3.16±0.01 | 3.86±0.01 | 3.13±0.01 | 1.43±0.01 |
| *β*-Bisabolene | 1494 | 1729 | 0.33±0.01 | 1.23±0.01 | 0.39±0.01 | 0.56±0.03 | 3.26±0.01 |
| Bicyclogermacrene | 1500 | 1718 | 0.21±0.01 | - | 0.47±0.01 | 0.33±0.01 | 0.86±0.01 |
| *β* -Curcumene | 1514 | 1731 | 6.11±0.01 | 3.84±0.01 | - | - | - |
| *δ*-Cadinene | 1517 | 1745 | - | - | 0.82±0.03 | 0.72±0.01 | 0.84±0.05 |
| **OS** | | | **11.75** | **7.12** | **39.39** | **38.25** | **21.24** |
| Spathulenol* | 1577 | 2101 | - | - | 5.14±0.01 | 4.91±0.01 | 1.66±0.01 |
| Caryophyllene oxide* | 1581 | 1955 | 9.32±0.01 | 5.53±0.01 | 3.95±0.01 | 5.97±0.01 | 4.83±0.01 |
| *γ*-Eudesmol | 1632 | 2135 | 1.14±0.01 | 0.96±0.07 | 1.22±0.01 | 0.89±0.01 | 0.68±0.03 |
| *α*-Cadinol | 1655 | 2208 | 0.82±0.01 | 0.22±0.01 | 0.54±0.01 | 0.91±0.01 | - |
| *α*-Bisabolol | 1688 | 2116 | 0.26±0.01 | 0.41±0.1 | 25.78±0.01 | 23.02±0.01 | 11.92±0.01 |

| | | | | | | | |
|---|---|---|---|---|---|---|---|
| *α*-Bisabolol oxide | 1748 | 2511 | 0.21±0.01 | - | 2.76±0.01 | 2.55±0.02 | 2.15±0.01 |
| **PC** | | | - | **1.16** | **2.64** | **1.37** | **4.27** |
| Thymol* | 1290 | 2198 | - | - | 0.88±0.01 | - | 0.84±0.01 |
| Carvacrol* | 1298 | 2239 | - | 1.16±0.05 | 0.31±0.05 | 0.72±0.01 | 3.43±0.01 |
| Eugenol | 1370 | 2175 | - | - | 0.45±0.01 | 0.65±0.01 | - |
| **CC** | | | - | **0.65** | **1.93** | **2.56** | **1.99** |
| 1-Octen-3-*ol* | 974 | 1433 | - | - | - | - | 0.28±0.07 |
| *β*-Ionone | 1487 | 1924 | - | 0.65±0.01 | 1.93±0.01 | 2.56±0.01 | 1.71±0.01 |
| **H** | | | **0.81** | **0.31** | **1.99** | **3.42** | **4.34** |
| Eicosane* | 2000 | 2000 | - | 0.31±0.01 | - | 0.25±0.01 | 0.45±0.01 |
| Docosane* | 2200 | 2200 | 0.81±0.01 | - | - | 1.46±0.01 | 1.32±0.01 |
| Tricosane* | 2300 | 2300 | - | - | - | 0.72±0.01 | - |
| Tetracosane* | 2400 | 2400 | - | - | - | - | 0.66±0.01 |
| Pentacosane* | 2500 | 2500 | - | - | 0.35±0.01 | 0.42±0.03 | 1.35±0.01 |
| Hexacosane* | 2600 | 2600 | - | - | 0.73±0.01 | 0.57±0.01 | - |
| Heptacosane* | 2700 | 2700 | - | - | 0.45±0.03 | - | - |
| Octacosane* | 2800 | 2800 | - | - | 0.24±0.01 | - | 0.56±0.01 |
| Nonacosane* | 2900 | 2900 | - | - | 0.22±0.01 | - | - |
| ***Total identified (%)*** | | | **95.24** | **95.89** | **93.04** | **90.37** | **95.37** |

Retention indices were determined relative to a series of *n*-alkanes ($C_8$–$C_{40}$) on capillary columns VF5-ms (RI[a]) and CP Wax 52 (RI[b]); identification method: RI comparison of RIs with those listed in a homemade library; reported in the literature [87] and/or authentic samples; comparison of mass spectra with those in mass spectral libraries NIST02 [88] and Wiley 9; *, injection reference com-

pounds; SD, standard deviation; MH, Monoterpene hydrocarbons; OM, Oxygenated monoterpenes; SH, Sesquiterpene hydrocarbons; OS, Oxygenated sesquiterpenes; PC, Phenolic compounds; CC, Carbonylic compounds; H, Hydrocarbons.
