# Supplementary material for: Phytochemicals and Their Correlation with Molecular Data in Micromeria and Clinopodium (Lamiaceae) Taxa
Source: Plants (Basel). 2022 Dec 6;11(23):3407. doi: 10.3390/plants11233407 (PMC9739532; doi:10.3390/plants11233407)
Supplement: Supplementary file 1 [file plants-11-03407-s001.zip › Table S4.pdf]

plants

Table S4. Phytochemical composition, identification and major groups of chemical components (%) of essential oil (EO) of *Micromeria juliana* (Mj).

			Sample and yield									
			Mj1	Mj2	Mj3	Mj4	Mj5	Mj6	Mj7	Mj8	Mj9	Mj10
			0.96	1.02	0.98	1.02	0.92	1.16	0.94	1.34	1.03	0.98
Component	RI[a]	RI[b]	EO±SD	EO±SD	EO±SD	EO±SD	EO±SD	EO±SD	EO±SD	EO±SD	EO±SD	EO±SD
**MH**			**5.61**	**9.57**	**6.23**	**7.52**	**8.29**	**9.55**	**7.38**	**7.51**	**10.5**	**10.69**
α-Thujene	924	1029	-	1.42±0.01	-	3.53±0.01	4.72±0.01	7.31±0.01	-	-	-	-
α-Pinene*	938	1025	0.54±0.01	0.66±0.03	0.43±0.03	0.92±0.01	0.23±0.03	0.21±0.01	1.62±0.01	1.13±0.01	1.52±0.01	1.66±0.01
Verbenene	960	1121	-	0.44±0.01	-	-	0.48±0.01	0.32±0.01	0.54±0.01	0.22±0.01	0.41±0.01	0.85±0.01
Camphene*	962	1056	0.75±0.01	0.21±0.03	0.72±0.01	0.38±0.02	0.42±0.01	0.31±0.03	0.42±0.01	0.21±0.03	1.13±0.01	1.12±0.01
Sabinene	971	1126	0.38±0.01	0.65±0.01	0.31±0.03	-	1.14±0.01	0.93±0.01	-	-	0.92±0.01	0.46±0.01
β-Pinene	982	1092	1.08±0.01	1.32±0.01	0.98±0.01	1.93±0.01	0.66±0.01	0.21±0.01	1.63±0.01	3.23±0.01	1.12±0.01	1.59±0.01

Myrcene	992	1173	-	-	-	-	0.31±0.01	0.12±0.01	-	-	-	-
δ-3-Carene	1008	1147	-	-	-	-	-	-	0.64±0.01	0.41±0.03	-	-
α-Terpinene	1016	1192	-	1.25±0.03	1.02±0.01	-	-	-	0.35±0.01	0.62±0.01	-	0.74±0.01
p-Cymene	1021	1270	-	-	-	-	-	-	0.32±0.01	0.21±0.01	-	-
β-Phellandrene	1025	1208	-	-	-	-	-	-	1.02±0.01	1.15±0.01	-	-
Limonene	1032	1204	2.58±0.01	3.62±0.01	2.52±0.01	0.76±0.01	0.33±0.01	0.14±0.01	0.84±0.02	0.33±0.01	1.25±0.03	2.02±0.01
γ-Terpinene	1057	1225	0.28±0.01	-	0.25±0.03	-	-	-	-	-	-	-
cis-Sabinene hydrate	1065	1456	-	-	-	-	-	-	-	-	4.15±0.01	2.25±0.01
**OM**			**15.02**	**14.59**	**17.30**	**16.04**	**13.78**	**10.42**	**17.78**	**16.21**	**18.31**	**17.00**
trans-Linalool oxide *	1088	1434	0.23±0.01	-	0.35±0.01	0.21±0.01	0.46±0.01	0.41±0.03	0.46±0.01	0.73±0.01	0.23±0.01	1.43±0.01
Linalool*	1099	1548	0.65±0.01	0.45±0.01	0.56±0.01	0.41±0.02	0.27±0.03	0.61±0.01	1.33±0.01	1.21±0.03	4.52±0.01	2.52±0.01
α-Campholenal	1129	1496	-	-	-	-	-	-	0.46±0.01	0.65±0.01	-	-
Isopulegol	1145	-	-	0.83±0.01	-	-	-	-	-	-	-	-

trans-Pinocarveol	1147	1658	0.86±0.03	0.26±0.01	-	0.31±0.01	0.33±0.01	-	-	0.32±0.01	0.23±0.01	0.85±0.01
Menthone	1148	1462	0.93±0.01	0.43±0.03	0.18±0.01	-	-	-	-	-	-	-
Camphor	1151	1499	-	-	0.33±0.01	-	-	-	0.43±0.01	0.82±0.01	-	-
Isoborneol	1155	1649	-	-	-	-	-	-	0.25±0.03	0.51±0.01	-	-
Pinocarvone	1160	1565	-	-	-	-	-	-		-	0.62±0.01	-
Borneol*	1176	1699	1.61±0.01	1.43±0.01	1.75±0.01	1.43±0.01	1.21±0.01	0.83±0.01	1.21±0.01	1.11±0.03	0.35±0.01	2.35±0.01
Terpinen-4-*ol*	1184	1601	-	-	-	-	-	-	-	-	-	0.41±0.01
α-Terpineol	1186	1686	0.47±0.05	0.32±0.01	0.56±0.01	0.81±0.01	0.52±0.01	0.12±0.01	0.71±0.01	0.13±0.01	0.14±0.03	0.14±0.03
Myrtenol	1197	1782	-	-	0.85±0.05	-	0.49±0.02	0.23±0.07	-	0.41±0.03	0.21±0.01	0.21±0.01
Verbenone	1204	1705	9.29±0.01	10.29±0.01	10.24±0.01	9.83±0.03	8.31±0.01	6.63±0.01	11.21±0.01	9.33±0.01	11.2±0.01	6.72±0.01
trans-Carveol	1215	1815	0.98±0.01	0.58±0.01	0.29±0.05	0.63±0.02	0.58±0.01	0.43±0.01	0.29±0.03	0.34±0.01	-	0.76±0.01
Piperitone	1250	1719	-	-	-	-	-	-	-	-	0.81±0.03	1.61±0.01
Bornyl acetate	1285	1570	-	-	1.28±0.03	1.54±0.01	1.29±0.01	0.65±0.03	-	-	-	-

α-Terpenyl acetate	1349	1685	-	-	0.91±0.01	-	-	-	0.51±0.01	0.34±0.03	-	-
Neryl acetate	1358	1692	-	-	-	0.87±0.01	0.32±0.01	0.51±0.01	0.92±0.01	0.31±0.01	-	-
**SH**			**31.31**	**35.56**	**28.28**	**27.01**	**21.25**	**17.72**	**26.21**	**22.71**	**26.84**	**33.05**
α-Copaene	1377	1484	0.86±0.01	0.26±0.03	0.15±0.01	0.61±0.01	1.32±0.02	0.21±0.01	0.77±0.01	0.75±0.01	0.54±0.01	0.85±0.01
β-Bourbonene	1383	1508	0.59±0.05	-	0.34±0.01	0.22±0.01	0.71±0.01	0.42±0.01	1.75±0.01	1.14±0.03	0.21±0.01	0.65±0.01
β-Elemene	1389	1593	-	0.54±0.01	-	-	-	-	0.38±0.01	1.31±0.01	-	-
E-Caryophyllene*	1424	1585	20.04±0.01	22.35±0.01	16.91±0.01	17.75±0.01	12.35±0.01	10.62±0.01	14.78±0.01	11.15±0.01	17.91±0.03	19.75±0.01
β-Copaene	1429	1584	0.97±0.01	1.54±0.01	1.34±0.02	0.63±0.01	-	0.73±0.01	0.95±0.01	0.51±0.01	0.85±0.01	0.45±0.01
trans-*α*-Bergamotene	1433	1580	-	-	0.21±0.01	-	0.47±0.01	0.13±0.01	-	-	0.23±0.01	0.28±0.01
α-Humulene	1456	1654	0.78±0.01	1.72±0.02	2.23±0.01	-	0.42±0.01	-	0.86±0.01	1.92±0.01	1.13±0.01	1.26±0.01
allo-Aromadendrene*	1465	1662	2.28±0.01	1.25±0.01	1.95±0.01	2.72±0.01	-	1.23±0.01	-	-	0.52±0.05	1.23±0.01
β-Chamigrene	1477	1735	-	-	-	-	-	-	0.37±0.01	0.32±0.01	0.21±0.01	0.63±0.03
Germacrene D	1481	1692	4.36±0.01	4.62±0.01	5.15±0.05	4.26±0.01	4.72±0.01	3.12±0.01	3.53±0.01	2.76±0.06	3.32±0.01	4.83±0.01

β-Bisabolene	1494	1729	1.43±0.01	0.63±0.01	-	0.82±0.01	1.26±0.02	1.26±0.07	0.83±0.01	0.75±0.01	0.56±0.01	0.84±0.01
Viridiflorene	1496	1697	-	1.02±0.01	-	-	-	-	0.63±0.01	0.82±0.01	-	-
β-Curcumene	1514	1731	-	-	-	-	-	-	0.63±0.01	0.86±0.01	-	-
δ-Cadinene	1517	1745	0.83±0.01	1.63±0.01	-	-	-	-	0.73±0.01	0.42±0.03	1.36±0.01	2.28±0.01
**OS**			**31.12**	**27.07**	**35.14**	**28.97**	**30.54**	**31.93**	**31.32**	**36.05**	**32.59**	**26.18**
Spathulenol*	1577	2101	3.05±0.01	2.13±0.01	3.45±0.01	3.84±0.01	3.91±0.01	4.81±0.01	-	-	-	0.91±0.01
Caryophyllene oxide*	1581	1955	26.04±0.01	24.34±0.01	30.42±0.01	22.26±0.01	25.73±0.01	25.5±0.01	30.61±0.01	32.72±0.01	29.3±0.01	23.91±0.01
γ-Eudesmol	1632	2135	0.72±0.01	0.12±0.02	0.32±0.01	1.13±0.01	0.62±0.02	0.92±0.01	0.71±0.01	0.67±0.01	0.64±0.01	0.44±0.01
α-Muurolol	1645	2163	-	-	-	-	-	-	-	-	0.52±0.01	0.51±0.01
α-Cadinol	1655	2208	0.84±0.01	0.14±0.01	0.42±0.03	0.82±0.01	-	0.23±0.01	-	-	0.65±0.01	0.41±0.01
α-Bisabolol	1688	2116	0.47±0.03	0.34±0.01	0.53±0.01	0.92±0.01	0.28±0.01	0.47±0.01	-	2.34±0.01	0.92±0.01	-
α-Bisabolol oxide	1748	2511	-	-	-	-	-	-	-	0.32±0.01	0.56±0.01	-
**PC**			**-**	**2.54**	**2.24**	**2.84**	**4.17**	**9.46**	**4.8**	**4.48**	**3.52**	**2.51**

Thymol*	1290	2198	-	2.54±0.01	1.51±0.01	2.26±0.01	3.84±0.01	8.33±0.01	3.59±0.01	2.54±0.03	3.52±0.01	2.51±0.01
Carvacrol*	1298	2239	-	-	0.73±0.01	0.58±0.01	0.33±0.01	1.13±0.01	1.21±0.01	1.94±0.01	-	-
Eugenol	1370	2175	-	-	-	-	-	-		-	-	-
**CC**			**0.55**	**0.73**	**0.79**	**1.01**	**1.02**	**0.36**	**1.59**	**1.50**	**1.36**	**1.00**
1-Octen-3-*ol*	974	1433	-	-	-	-	-	-	0.68±0.01	1.22±0.01	0.91±0.01	0.42±0.01
3-Octanol acetate	1125	1376	-	-	0.34±0.02	0.48±0.01	-	-	-	-	0.11±0.01	0.36±0.01
Isobutyl hexanoate	1155	1356	-	-	-	-	-	-	-	-	0.34±0.01	0.22±0.01
β-Ionone	1487	1924	0.55±0.01	0.73±0.01	0.45±0.01	0.53±0.05	1.02±0.01	0.36±0.01	0.91±0.03	0.28±0.01	-	-
**H**			**8.44**	**5.97**	**4.48**	**10.55**	**14.07**	**12.10**	**7.43**	**5.89**	**1.76**	**6.42**
Eicosane*	2000	2000	-	0.73±0.01	1.55±0.01	0.88±0.01	0.45±0.01	0.33±0.01	0.56±0.01	0.48±0.01	0.28±0.01	0.44±0.01
Heneicosane*	2100	2100	-	-	-	-	-	-	-	0.67±0.01	-	-
Docosane*	2200	2200	7.57±0.01	4.57±0.01	2.51±0.01	9.08±0.01	11.53±0.03	11.28±0.01	5.75±0.01	3.55±0.01	-	3.95±0.01
Tricosane*	2300	2300	-	-	0.42±0.01	-	-	-	-	-	-	-

Tetracosane*	2400	2400	-	-	-	-	-	-	-	-	0.32±0.01	0.72±0.01
Pentacosane*	2500	2500	0.87±0.01	-	-	0.35±0.01	1.33±0.01	0.34±0.01	0.32±0.01	0.16±0.01	0.52±0.01	0.39±0.01
Hexacosane*	2600	2600	-	0.67±0.01	-	-	-	-	-	-	0.64±0.01	0.92±0.01
Heptacosane*	2700	2700	-	-	-	-	-	-	0.48±0.01	0.21±0.01	-	-
Octacosane*	2800	2800	-	-	-	0.24±0.01	0.76±0.01	0.15±0.01	-	-	-	-
Nonacosane*	2900	2900	-	-	-	-	-	-	0.32±0.01	0.82±0.01	-	-
Total identified (%)			**92.05**	**96.03**	**94.46**	**93.94**	**93.12**	**91.54**	**96.51**	**94.35**	**94.88**	**96.85**

Retention indices were determined relative to a series of *n*-alkanes (C_8–C_{40}) on capillary columns VF5-ms (RI[a]) and CP Wax 52 (RI[b]); identification method: RI comparison of RIs with those listed in a homemade library; reported in the literature [87] and/or authentic samples; comparison of mass spectra with those in mass spectral libraries NIST02 [88] and Wiley 9; *, injection reference compounds; SD, standard deviation; MH, Monoterpene hydrocarbons; OM, Oxygenated monoterpenes; SH, Sesquiterpene hydrocarbons; OS, Oxygenated sesquiterpenes; PC, Phenolic compounds; CC, Carbonylic compounds; H, Hydrocarbons.
