# Supplementary material for: Phytochemicals and Their Correlation with Molecular Data in Micromeria and Clinopodium (Lamiaceae) Taxa
Source: Plants (Basel). 2022 Dec 6;11(23):3407. doi: 10.3390/plants11233407 (PMC9739532; doi:10.3390/plants11233407)
Supplement: Supplementary file 1 [file plants-11-03407-s001.zip › Table S5.pdf]

plants

Table S5. Phytochemical composition, identification and major groups of chemical components (%) of essential oil (EO) of *Micromeria kerneri* (Mk).

			Sample and yield				
			Mk1	Mk2	Mk3	Mk4	Mk5
			0.73	0.76	0.77	0.75	0.62
Component	RI[a]	RI[b]	EO±SD	EO±SD	EO±SD	EO±SD	EO±SD
**MH**			**32.74**	**35.69**	**35.59**	**37.68**	**44.73**
α-Pinene*	938	1025	12.3±0.03	14.31±0.01	12.62±0.01	15.43±0.01	16.13±0.01
Verbenene	960	1121	2.15±0.01	2.13±0.01	0.26±0.03	2.13±0.01	2.32±0.01
Camphene*	962	1056	0.62±0.03	-	0.25±0.01	-	0.44±0.01
β-Pinene	982	1092	12.10±0.01	10.65±0.01	13.44±0.01	10.65±0.01	13.12±0.01
Myrcene*	992	1173	0.24±0.03	-	-	0.38±0.01	0.24±0.01
β-Phellandrene	1025	1208	5.21±0.07	5.23±0.01	5.11±0.01	5.86±0.01	6.93±0.01
Limonene	1032	1204	0.12±0.03	2.51±0.01	2.35±0.01	3.23±0.01	3.12±0.01
γ-Terpinene	1057	1225	-	0.86±0.01	1.56±0.01	-	2.43±0.02
**OM**			**13.34**	**12.14**	**17.21**	**13.36**	**22.45**
trans-Linalool oxide*	1088	1434	1.13±0.01	-	1.76±0.01	-	0.84±0.01
Linalool*	1099	1548	1.31±0.05	1.32±0.01	1.93±0.01	1.86±0.01	1.33±0.03
α-Campholenal	1129	1496	-	-	0.45±0.01	-	5.25±0.03
trans-Pinocarveol	1147	1658	0.23±0.01	-	0.75±0.01	0.56±0.01	0.22±0.01
Camphor	1151	1499	-	-	-	0.84±0.01	0.58±0.01

Pinocarvone	1160	1565	-	1.18±0.05	-	-	1.94±0.01
Borneol*	1176	1699	2.41±0.01	2.45±0.01	2.26±0.01	2.07±0.01	2.17±0.01
Terpinen-4-*ol*	1184	1601	0.22±0.03	-	-	-	-
α-Terpineol	1186	1686	0.31±0.01	0.54±0.01	0.44±0.01	0.74±0.01	0.51±0.01
Myrtenol	1197	1782	0.33±0.01	0.56±0.01	3.54±0.01	0.81±0.01	4.25±0.01
Verbenone	1204	1705	3.73±0.02	2.55±0.01	2.61±0.01	2.35±0.01	1.51±0.01
trans-Carveol	1215	1815	0.53±0.01	-	0.21±0.01	-	0.74±0.01
endo-Fenchyl acetate	1218	1465	-	-	0.63±0.01	-	-
Pulegone	1234	1641	-	0.91±0.01	-	0.87±0.01	0.56±0.01
Piperitone	1250	1719	3.14±0.03	2.63±0.01	2.12±0.01	3.26±0.01	2.32±0.01
Piperitenone oxide	1366	1941	-	-	0.51±0.03	-	0.23±0.01
**SH**			**22.64**	**13.08**	**16.69**	**16.25**	**8.65**
α-Copaene	1377	1484	0.23±0.01	0.15±0.01	0.36±0.01	0.75±0.01	0.66±0.01
β-Bourbonene	1383	1508	0.31±0.01	0.35±0.03	0.91±0.01	0.51±0.01	0.47±0.01
β-Elemene	1389	1593	-	-	-	0.41±0.01	0.51±0.01
E-Caryophyllene*	1424	1585	8.31±0.03	4.19±0.01	6.52±0.01	1.52±0.01	1.35±0.01
β-Copaene	1429	1584	1.13±0.01	1.18±0.01	0.67±0.01	0.45±0.05	0.25±0.01
α-Humulene	1456	1654	1.24±0.01	-	1.13±0.01	0.95±0.01	-
allo-Aromadendrene*	1465	1662	4.81±0.01	1.96±0.01	3.31±0.01	3.23±0.01	1.63±0.01
Germacrene D*	1481	1692	1.92±0.01	2.16±0.01	1.77±0.01	6.24±0.01	2.31±0.01
β-Bisabolene	1494	1729	0.96±0.05	0.61±0.01	0.81±0.01	0.64±0.01	0.99±0.01

Viridiflorene	1496	1697	2.12±0.01	1.05±0.01	1.21±0.01	1.01±0.01	0.48±0.01
δ-Cadinene	1517	1745	1.61±0.01	1.43±0.01	-	0.54±0.03	-
**OS**			**21.94**	**27.41**	**20.37**	**20.07**	**14.77**
Spathulenol*	1577	2101	0.11±0.01	2.46±0.01	2.93±0.01	1.71±0.01	1.10±0.01
Caryophyllene oxide*	1581	1955	17.32±0.01	23.46±0.01	14.42±0.01	14.32±0.01	12.81±0.01
γ-Eudesmol	1632	2135	2.12±0.01	0.32±0.01	2.28±0.01	2.83±0.01	0.86±0.03
α-Cadinol	1655	2208	0.12±0.01	0.43±0.01	-	-	-
α-Bisabolol	1688	2116	0.34±0.01	0.31±0.01	0.74±0.01	0.35±0.01	-
α-Bisabolol oxide	1748	2511	1.93±0.01	0.43±0.01	-	0.86±0.01	-
**PC**			**2.41**	**0.53**	**0.39**	**1.66**	-
Thymol*	1290	2198	2.26±0.01	0.53±0.05	-	1.35±0.01	-
Carvacrol*	1298	2239	0.15±0.01	-	0.21±0.01	0.31±0.07	-
Eugenol*	1370	2175	-	-	0.18±0.05		-
**CC**			**0.38**	**0.75**	**0.98**	**1.99**	**1.43**
1-Octen-*3-ol*	974	1433	0.16±0.01	0.53±0.01	0.32±0.02	1.43±0.01	1.12±0.01
3-Octanol acetate	1125	1376	0.22±0.01	-	0.45±0.01	0.42±0.01	-
Isobutyl hexanoate	1155	-	-	-	0.21±0.03	-	-
β-Ionone	1487	1924	-	0.22±0.01	-	0.14±0.01	0.32±0.01
**H**			**1.61**	**1.71**	**1.51**	**0.97**	**2.06**
Eicosane*	2000	2000	0.23±0.01	-	-	-	0.64±0.01
Docosane*	2200	2200	-	0.72±0.01	0.31±0.05	0.55±0.01	1.42±0.03

Tricosane*	2300	2300	0.42±0.01	0.47±0.01	0.24±0.01	0.42±0.05	-
Tetracosane*	2400	2400	0.44±0.01	-	0.51±0.01	-	-
Pentacosane*	2500	2500	-	-	-	-	-
Hexacosane*	2600	2600	0.52±0.01	0.52±0.01	0.45±0.01	-	-
Total identified (%)			**95.06**	**91.31**	**92.74**	**91.98**	**94.09**

Retention indices were determined relative to a series of *n*-alkanes (C_8–C_{40}) on capillary columns VF5-ms (RI^a) and CP Wax 52 (RI^b); identification method: RI comparison of RIs with those listed in a homemade library; reported in the literature [] and/or authentic samples; comparison of mass spectra with those in mass spectral libraries NIST02 [88] and Wiley 9; *, injection reference compounds; SD, standard deviation; MH, Monoterpene hydrocarbons; OM, Oxygenated monoterpenes; SH, Sesquiterpene hydrocarbons; OS, Oxygenated sesquiterpenes; PC, Phenolic compounds; CC, Carbonylic compounds; H, Hydrocarbons.
