# Supplementary material for: Phytochemicals and Their Correlation with Molecular Data in Micromeria and Clinopodium (Lamiaceae) Taxa
Source: Plants (Basel). 2022 Dec 6;11(23):3407. doi: 10.3390/plants11233407 (PMC9739532; doi:10.3390/plants11233407)
Supplement: Supplementary file 1 [file plants-11-03407-s001.zip › Table S6.pdf]

plants MDPI

Table S6. Phytochemical composition, identification and major groups of chemical components (%) of essential oil (EO) of *Micromeria longipedunculata* (Ml) and *M. microphylla* (Mm).

			Sample and yield				
			Ml1	Ml2	Ml3	Ml4	Mm
			0.95	0.92	1.0	0.71	0.71
Component	RI[a]	RI[b]	EO±SD	EO±SD	EO±SD	EO±SD	EO±SD
**MH**			**5.81**	**4.52**	**3.24**	**10.36**	**20.56**
α-Pinene*	938	1025	0.53±0.03	1.21±0.01	0.84±0.03	0.93±0.01	2.62±0.01
Verbenene	960	1121	-	-	-	-	0.26±0.03
Camphene*	962	1056	-	-	-	-	0.45±0.01
β-Pinene	982	1092	0.62±0.01	0.98±0.01	-	1.17±0.03	7.14±0.03
Myrcene	992	1173	0.36±0.01	0.42±0.01	0.31±0.01	0.72±0.01	2.24±0.01
α-Terpinene	1016	1192	-	-	-	-	0.73±0.01
p-Cymene	1021	1270	-	-	-	-	1.13±0.01
β-Phellandrene	1025	1208	-	-	-	-	1.51±0.01
Limonene	1032	1204	3.25±0.01	1.03±0.05	0.85±0.01	6.24±0.01	2.33±0.01
(Z)-β-Ocimene*	1052	1218	0.82±0.01	0.88±0.01	1.24±0.01	1.14±0.01	-
γ-Terpinene	1057	1225	-	-	-	-	1.91±0.01
allo-Ocimene	1128	1370	0.23±0.01	-	-	0.16±0.01	0.24±0.01
**OM**			**30.80**	**30.73**	**35.89**	**30.13**	**16.89**
trans-Linalool oxide*	1088	1434	-	-	-	-	3.46±0.01
Linalool*	1099	1548	1.76±0.01	2.93±0.01	2.56±0.01	2.44±0.01	0.93±0.01

β-Thujone	1121	1438	0.22±0.01	0.74±0.03	0.32±0.01	0.82±0.01	0.32±0.01
trans-Pinocarveol	1147	1658	1.56±0.01	2.02±0.01	0.76±0.01	0.37±0.01	0.45±0.01
Menthone	1148	1462	0.85±0.01	0.48±0.01	0.64±0.01	1.16±0.01	-
Camphor	1151	1499	0.34±0.01	-	0.55±0.03	-	-
Borneol*	1176	1699	0.37±0.01	-	-	0.28±0.01	1.26±0.01
Terpinen-4-*ol*	1184	1601	-	0.28±0.01	-	0.34±0.01	2.74±0.01
α-Terpineol	1186	1686	0.24±0.01	-	0.75±0.07	-	0.41±0.01
Myrtenol	1197	1782	0.71±0.01	1.04±0.01	0.33±0.03	0.25±0.01	2.54±0.01
trans-Carveol	1215	1815	-	-	0.28±0.01	-	1.91±0.01
endo-Fenchyl acetate	1218	1465	-	-	-	-	0.63±0.01
Pulegone	1234	1641	4.87±0.01	8.45±0.01	6.41±0.01	7.25±0.01	-
Piperitone	1250	1719	2.23±0.01	1.78±0.01	2.94±0.01	2.21±0.01	1.12±0.01
Bornyl acetate	1285	1570	0.35±0.01	-	0.36±0.01	-	0.34±0.03
Piperitonene	1340	1882	17.3±0.01	13.01±0.01	19.53±0.01	13.87±0.01	-
α-Terpenyl acetate	1349	1685	-	-	-	-	0.25±0.01
Neryl acetate	1358	1692	-	-	-	-	0.53±0.01
Piperitenone oxide	1366	1941	-	-	0.46±0.01	1.14±0.01	-
**SH**			**16.43**	**18.01**	**20.24**	**10.56**	**17.37**
α-Copaene	1377	1484	0.95±0.01	-	-	0.32±0.01	0.31±0.01
β-Bourbonene	1383	1508	0.51±0.01	1.04±0.01	-	0.91±0.01	0.91±0.01
β-Elemene	1389	1593	2.71±0.01	3.56±0.01	3.82±0.01	1.24±0.01	-

α-Gurjunene	1407	1520	-	-	-	-	0.25±0.01
E-Caryophyllene*	1424	1585	1.22±0.01	2.97±0.01	3.45±0.01	2.73±0.01	6.32±0.01
β-Copaene	1429	1584	0.51±0.01	0.84±0.01	-	1.32±0.03	0.63±0.01
(*Z*)-*β*-Farnesene	1454	1639	0.47±0.01	0.44±0.05	-	0.14±0.01	0.41±0.01
α-Humulene	1456	1654	0.15±0.01	1.87±0.01	2.04±0.01	1.25±0.01	0.18±0.01
allo-Aromadendrene*	1465	1662	1.17±0.01	0.09±0.01	2.44±0.01	-	3.81±0.01
β-Chamigrene	1477	-	0.28±0.01	-	-	-	-
Germacrene D*	1481	1692	2.67±0.01	5.93±0.01	6.95±0.01	2.38±0.01	1.57±0.01
β-Bisabolene	1494	1729	1.24±0.01	0.78±0.01	0.33±0.01	0.27±0.01	0.41±0.01
Viridiflorene	1496	1697	1.51±0.01	0.49±0.01	-	-	1.32±0.01
Bicyclogermacrene	1500	1718	2.21±0.01	-	1.21±0.01	-	1.25±0.01
δ-Cadinene	1517	1745	0.83±0.01	-	-	-	-
**OS**			**38.90**	**36.74**	**33.63**	**37.12**	**32.63**
Spathulenol*	1577	2101	36.71±0.01	36.02±0.01	31.98±0.01	32.41±0.01	3.93±0.01
Caryophyllene oxide*	1581	1955	0.32±0.01	0.48±0.01	0.36±0.01	-	4.82±0.01
γ-Eudesmol	1632	2135	0.86±0.01	-	-	0.87±0.05	0.23±0.01
α-Cadinol	1655	2208	-	-	0.82±0.01	0.36±0.01	-
α-Bisabolol	1688	2116	0.35±0.01	0.24±0.01	0.26±0.01	1.16±0.01	0.74±0.03
Eudesm-7(11)-en-4-*ol*	1700	2251	-	-	-	-	22.91±0.01
α-Bisabolol oxide	1748	2511	0.66±0.01	-	0.21±0.01	2.32±0.01	-
**PC**			**0.84**	**1.70**	-	**0.10**	**2.81**

Thymol*	1290	2198	0.32±0.01	-	-	-	-
Carvacrol*	1298	2239	0.31±0.03	0.86±0.01	-	0.10±0.01	1.91±0.01
Eugenol*	1370	2175	0.21±0.01	0.84±0.01	-	-	0.90±0.01
**CC**			**0.67**	-	-	**0.92**	**0.66**
1-Octen-3-*ol*	974	1433	0.12±0.01	-	-	0.92±0.01	.
3-Octanol acetate	1125	1376	0.42±0.01	-	-	-	0.45±0.01
Isobutyl hexanoate	1155	-	-	-	-	-	0.21±0.03
β-Ionone	1487	1924	0.13±0.01	-	-	-	-
**H**			**0.71**	**1.98**	**0.89**	**2.71**	**6.73**
Eicosane*	2000	2000	-	-	-	0.21±0.03	-
Heneicosane*	2100	2100	-	-	-	0.12±0.01	-
Docosane*	2200	2200	0.35±0.01	-	0.89±0.01	1.14±0.01	4.41±0.01
Tricosane*	2300	2300	0.12±0.01	0.21±0.01	-	-	0.24±0.01
Tetracosane*	2400	2400	-	-	-	-	0.51±0.01
Pentacosane*	2500	2500	0.24±0.02	0.88±0.01	-	0.45±0.01	0.32±0.01
Hexacosane*	2600	2600	-	0.89±0.01	-	0.58±0.01	1.25±0.01
Total identified (%)			**94.16**	**93.68**	**93.89**	**91.90**	**97.65**

Retention indices were determined relative to a series of *n*-alkanes (C_8–C_{40}) on capillary columns VF5-ms (RI^a) and CP Wax 52 (RI^b); identification method: RI comparison of RIs with those listed in a homemade library; reported in the literature [87] and/or authentic samples; comparison of mass spectra with those in mass spectral libraries NIST02 [88] and Wiley 9; *, injection reference compounds; SD, standard deviation; MH, Monoterpene hydrocarbons; OM, Oxygenated monoterpenes; SH, Sesquiterpene hydrocarbons; OS, Oxygenated sesquiterpenes; PC, Phenolic compounds; CC, Carbonylic compounds; H, Hydrocarbons.
