# Supplementary material for: Phytochemicals and Their Correlation with Molecular Data in Micromeria and Clinopodium (Lamiaceae) Taxa
Source: Plants (Basel). 2022 Dec 6;11(23):3407. doi: 10.3390/plants11233407 (PMC9739532; doi:10.3390/plants11233407)
Supplement: Supplementary file 1 [file plants-11-03407-s001.zip › Table S7.pdf]

plants

MDPI

Table S7. Phytochemical composition, identification and major groups of chemical components (%) of essential oil (EO) of.
Clinopodium dalmaticum (Cd) including *Micromeria bulgarica* (CdB), and *C. frivaldszkyanum* (Cf).

			Sample and yield						
			Cd1	Cd2	CdB1	CdB2	CdB3	Cf1	Cf2
			1.17	0.9	0.9	1.15	0.87	0.35	0.44
Component	RI[a]	RI[b]	EO±SD	EO±SD	EO±SD	EO±SD	EO±SD	EO±SD	EO±SD
**MH**			**7.40**	**6.95**	**12.88**	**11.20**	**25.17**	**6.52**	**11.55**
α-Thujene	924	1029	0.12±0.01	-	-	-	-	-	-
α-Pinene*	938	1025	-	-	5.52±0.01	1.43±0.01	14.31±0.01	2.43±0.03	2.62±0.01
Verbenene	960	1121	-	-	-	-	-	-	0.27±0.01
Camphene*	962	1056	0.62±0.01	0.21±0.03	-	0.42±0.03	0.72±0.02	0.72±0.01	0.42±0.03
Sabinene	971	1126	0.41±0.02	0.65±0.03	0.43±0.05	-	0.31±0.02	0.31±0.03	-

β-Pinene	982	1092	1.35±0.01	1.32±0.01	-	0.32±0.01	4.53±0.01	-	1.16±0.03
Myrcene	992	1173	0.23±0.03	-	-	-	-	-	0.95±0.03
α-Terpinene	1016	1192	-	1.25±0.03	-	-	-	-	0.78±0.03
p-Cymene	1021	1270	-	-	-	-	-	-	1.13±0.06
β-Phellandrene	1025	1208	-	-	-	-	1.12±0.01	-	-
Limonene	1032	1204	4.34±0.01	3.52±0.03	6.42±0.01	5.35±0.01	1.73±0.01	2.81±0.01	2.31±0.03
γ-Terpinene	1057	1225	-	-	0.51±0.01	1.91±0.01	0.24±0.03	0.25±0.03	1.91±0.01
cis-Sabinene hydrate	1065	1456	0.33±0.01	-	-	1.54±0.01	2.21±0.01	-	-
allo-Ocimene	1128	1370	-	-	-	0.23±0.02	-	-	-
**OM**			**60.05**	**67.19**	**9.28**	**15.00**	**22.71**	**68.66**	**53.00**
trans-Linalool oxide *	1088	1434	-	-	0.75±0.02	3.41±0.01	0.32±0.02	0.35±0.01	3.42±0.01

Linalool*	1099	1548	-	-	-	0.41±0.02	0.52±0.01	0.56±0.03	0.91±0.01
cis-*p*-Menth-*2*-*en*-*1*-ol	1118	1600	-	-	-	0.31±0.01	-	-	-
β-Thujone	1121	1438	-	-	-	0.32±0.01	-	-	0.31±0.01
α-Campholenal	1129	1496	-	-	-	0.54±0.03	0.11±0.01	-	-
Isopulegol	1145	1551	0.71±0.02	0.73±0.01	-	-	-	-	-
trans-Pinocarveol	1147	1658	0.24±0.01	0.24±0.01	-	0.46±0.01	-	-	0.44±0.01
Menthone	1148	1462		0.43±0.03	-	-	-	12.8±0.01	9.23±0.03
Camphor	1151	1499	-	-	0.85±0.01	3.25±0.01	4.34±0.01	0.33±0.01	-
Pinocarvone	1160	1565	1.14±0.03	0.91±0.01	-	-	-	-	-
Borneol*	1176	1699	2.43±0.02	-	-	-	4.11±0.01	-	-
Terpinen-4-*ol*	1184	1601	0.26±0.01	2.91±0.03	0.88±0.01	0.73±0.01	1.61±0.05	1.63±0.01	2.79±0.01
α-Terpineol	1186	1686	0.35±0.01	0.32±0.01	0.96±0.07	0.44±0.01	0.53±0.01	0.56±0.01	0.43±0.01

Myrtenol	1197	1782	0.33±0.01	-	-	0.52±0.02	0.41±0.01	0.45±0.01	2.57±0.1
Verbenone	1204	1705	-	-	1.32±0.01	0.31±0.01	2.23±0.01	0.24±0.01	-
trans-Carveol	1215	1815	0.15±0.01	0.54±0.01	-	1.93±0.01	5.21±0.01	1.23±0.01	0.91±0.03
endo-Fenchyl acetate	1218	1465	-	-	-	0.61±0.03	-	-	0.61±0.01
Pulegone	1233	1641	13.35±0.01	25.14±0.03	-	-	-	47.2±0.01	29.52±0.01
Piperitone	1250	1719	3.15±0.01	0.93±0.01	4.52±0.01	1.11±0.05	0.32±0.01	0.31±0.01	1.12±0.05
Linalyl acetate	1252	1553	-	-	-	-	0.85±0.01	0.81±0.01	-
Bornyl acetate	1285	1570	-	-	-	0.30±0.05	1.22±0.02	1.28±0.03	0.35±0.03
Piperitonene	1340	1882	36.63±0.02	34.73±0.01	-	-	-	-	-
α-Terpenyl acetate	1349	1685	-	-	-	0.22±0.01	0.93±0.01	0.91±0.01	0.25±0.01

Neryl acetate	1358	1692	-	-	-	0.13±0.01	-	-	0.14±0.01
Piperitenone oxide	1366	1941	2.26±0.01	0.31±0.02	-	-	-	-	-
**SH**			**19.51**	**14.18**	**41.41**	**48.98**	**13.08**	**10.07**	**15.9**
α-Copaene	1377	1484	0.21±0.01	0.26±0.03	2.52±0.01	0.34±0.01	0.12±0.05	0.15±0.01	0.31±0.01
β-Bourbonene	1383	1508	0.32±0.01	-	-	0.91±0.02	0.32±0.01	0.34±0.01	0.93±0.01
β-Elemene	1389	1593	-	0.54±0.01	-	-	-	-	-
α-Gurjunene	1407	1520	-	-	-	0.15±0.01	0.93±0.01	0.93±0.03	0.11±0.02
E-Caryophyllene*	1424	1585	8.33±0.01	2.35±0.01	31.74±0.01	42.43±0.01	2.91±0.01	2.91±0.01	6.33±0.01
β-Copaene	1429	1584	1.14±0.01	1.51±0.01	0.63±0.01	0.63±0.01	1.15±0.01	1.14±0.06	0.63±0.01
trans-*α*-Bergamotene	1433	1580	-	-	-	0.44±0.01	0.22±0.01	0.21±0.01	0.42±0.03
(*Z*)-*β*-Farnesene	1454	1639	-	-	-	0.42±0.01	0.33±0.01	0.34±0.01	0.40±0.01

α-Humulene	1456	1654	1.21±0.01	1.22±0.03	-	0.13±0.01	-	-	0.12±0.01
al-lo-Aromadendrene*	1465	1662	-	-	-	1.82±0.01	1.94±0.01	0.95±0.01	1.82±0.01
β-Chamigrene	1477	1735	-	-	0.66±0.01	0.28±0.01	0.34±0.01	-	0.25±0.03
Germacrene D*	1481	1692	3.61±0.01	3.62±0.01	4.71±0.01	0.77±0.01	2.15±0.02	2.15±0.05	1.52±0.01
β-Bisabolene	1494	1729	0.93±0.03	0.93±0.01	-	0.43±0.01	0.21±0.01	-	0.43±0.01
Viridiflorene	1496	1697	2.12±0.01	2.12±0.01	-	-	1.10±0.03	-	1.38±0.01
Bicyclogermacrene	1500	1718	-	-	1.15±0.01	0.23±0.05	0.91±0.03	0.95±0.01	1.25±0.01
δ-Cadinene	1517	1745	1.64±0.02	1.63±0.01	-	-	0.42±0.01	-	-
**OS**			**3.98**	**4.04**	**5.28**	**4.38**	**17.22**	**4.12**	**7.68**
Spathulenol*	1577	2101	0.12±0.01	0.13±0.01	2.51±0.01	0.92±0.01	2.42±0.01	2.43±0.01	3.92±0.01
Caryophyllene oxide*	1581	1955	1.31±0.01	1.31±0.01	0.52±0.01	1.52±0.01	13.41±0.01	0.42±0.01	2.83±0.01

γ-Eudesmol	1632	2135	2.12±0.01	2.12±0.02	0.42±0.01	0.21±0.05	0.33±0.01	0.32±0.01	0.22±0.01
α-Cadinol	1655	2208	0.11±0.03	0.14±0.01 -	1.83±0.03	-	0.41±0.01	0.42±0.03	-
α-Bisabolol	1688	2116	0.32±0.05	0.34±0.01	-	1.73±0.01	0.52±0.05	0.53±0.01	0.71±0.01
α-Bisabolol oxide	1748	2511	-	-	-	-	0.13±0.01	-	-
**PC**			**0.12**	**-**	**25.73**	**9.13**	**12.24**	**2.24**	**6.07**
Thymol*	1290	2198	-	-	25.73±0.01	9.13±0.01	11.53±0.01	1.51±0.01	4.13±0.01
Carvacrol*	1298	2239	0.12±0.02	-	-	-	0.71±0.01	0.73±0.01	1.94±0.01
**CC**			**0.21**	**0.92**	**0.72**	**1.30**	**1.57**	**0.90**	**1.40**
1-Octen-3-*ol*	974	1433	-	-	0.72±0.01	-	0.72±0.01	-	-
3-Octanol acetate	1125	1376	0.21±0.01	-	-	-	0.21±0.02	0.24±0.03	0.44±0.01
Isobutyl hexanoate	1155	1356	-	-	-	0.56±0.01	-	-	0.22±0.01
Isoamylhexanoate	1256	1457	-	-	-	0.53±0.01	0.32±0.01	0.31±0.01	0.51±0.01
β-Ionone	1487	1924	-	0.92±0.01	-	0.21±0.01	0.32±0.01	0.35±0.01	0.23±0.01

**H**			**2.12**	**1.79**	**1.61**	**1.59**	**1.55**	**0.42**	**0.67**
Eicosane*	2000	2000	0.82±0.01	1.23±0.01	0.22±0.03	0.42±0.01	-	-	-
Heneicosane*	2100	2100	-	-	0.34±0.01	-	0.14±0.01	-	-
Docosane*	2200	2200	-	-	0.63±0.01	0.46±0.01	0.21±0.01	-	0.44±0.01
Tricosane*	2300	2300	0.44±0.01	-	0.42±0.01	0.71±0.01	0.42±0.01	0.42±0.01	0.23±0.01
Tetracosane*	2400	2400	0.45±0.02	0.34±0.01	-	-	-	-	-
Pentacosane*	2500	2500	-	-	-	-	0.21±0.01	-	-
Hexacosane*	2600	2600	0.41±0.01	0.22±0.01	-	-	-	-	-
Heptacosane*	2700	2700	-	-	-	-	0.31±0.01	-	-
Octacosane*	2800	2800	-	-	-	-	0.26±0.02	-	-
Total identified (%)			**93.39**	**95.07**	**96.91**	**91.58**	**93.54**	**92.93**	**96.27**

Retention indices were determined relative to a series of *n*-alkanes (C_8–C_{40}) on capillary columns VF5-ms (RI^a) and CP Wax 52 (RI^b); identification method: RI comparison of RIs with those listed in a homemade library; reported in the literature [87] and/or authentic samples; comparison of mass spectra with those in mass spectral libraries NIST02 [88] and Wiley 9; *, injection reference compounds; SD, standard deviation; MH, Monoterpene hydrocarbons; OM, Oxygenated monoterpenes; SH, Sesquiterpene hydrocarbons; OS, Oxygenated sesquiterpenes; PC, Phenolic compounds; CC, Carbonylic compounds; H, Hydrocarbons.
