# Supplementary material for: Phytochemicals and Their Correlation with Molecular Data in Micromeria and Clinopodium (Lamiaceae) Taxa
Source: Plants (Basel). 2022 Dec 6;11(23):3407. doi: 10.3390/plants11233407 (PMC9739532; doi:10.3390/plants11233407)
Supplement: Supplementary file 1 [file plants-11-03407-s001.zip › Table S8.pdf]

plants

MDPI

Table S8. Phytochemical composition, identification and major groups of chemical components (%) of essential oil (EO) of *Clinopodium pulegium* (Cp), *C. serpyllifolium* (Cs), and *C. thymifolium* (Ct).

			Sample and yield								
			Cp	Cs	Ct1	Ct2	Ct3	Ct4	Ct5	Ct6	Ct7
			0.83	0.91	1.00	1.05	1.10	1.12	0.98	0.90	1.20
Component	RI[a]	RI[b]	EO±SD	EO±SD	EO±SD	EO±SD	EO±SD	EO±SD	EO±SD	EO±SD	EO±SD
**MH**			**16.17**	**8.31**	**5.61**	**6.31**	**2.55**	**3.15**	**3.83**	**2.92**	**2.20**
α-Thujene	924	1029	-	-	1.35±0.01	1.15±0.01	0.65±0.03	0.79±0.01	1.15±0.01	0.86±0.01	1.05±0.01
α-Pinene*	938	1025	1.22±0.03	0.52±0.02	0.72±0.01	0.36±0.01	-	-	0.93±0.01	-	-
Verbenene	960	1121	-	-	0.42±0.01	2.17±0.01	0.43±0.01	-	-	0.43±0.05	-
Camphene*	962	1056	0.24±0.01	-	0.44±0.01	0.61±0.03	-	-	-	-	-
Sabinene	971	1126	3.25±0.01	1.43±0.01	-	-	-	-	-	-	-
β-Pinene	982	1092	-	-	1.12±0.01	1.30±0.01	1.22±0.01	1.38±0.01	1.07±0.03	1.63±0.03	1.15±0.01
Myrcene	992	1173	-	-	0.24±0.01	0.23±0.03	-	-	0.52±0.01	-	-

α-Terpinene	1016	1192	0.64±0.01	-	-	-	0.25±0.01	-	-	-	-
Limonene	1032	1204	5.62±0.01	6.12±0.02	1.32±0.01	0.13±0.01	-	-	-	-	-
(*Z*)-*β*-Ocimene*	1052	1218	0.61±0.01	0.13±0.01	-	-	-	-	-	-	-
γ-Terpinene	1057	1225	0.22±0.01	0.11±0.01	-	-	-	-	-	-	-
allo-Ocimene	1128	1370	0.24±0.01	-	-	0.36±0.01	-	0.98±0.01	0.16±0.01	-	-
**OM**			**68.67**	**64.99**	**82.04**	**69.40**	**73.64**	**82.26**	**81.1**	**84.07**	**85.31**
trans-Linalool oxide *	1088	1434	-	-	0.74±0.01	1.14±0.01	0.24±0.01	0.36±0.01	0.32±0.01	0.21±0.01	0.41±0.01
Linalool*	1099	1548	2.64±0.01	-	0.36±0.01	0.34±0.03	-	-	-	-	-
cis-*p*-Menth-*2*-*en*-*1*-ol	1118	1600	-	-	1.31±0.01	1.65±0.01	2.12±0.01	1.42±0.01	1.36±0.01	1.72±0.01	0.98±0.01
α-Campholenal	1129	1496	-	-	-	0.38±0.01	-	-	-	-	-
Isopulegol	1145	1551	-	1.34±0.01	-	0.65±0.05	-	-	-	-	-
trans-Pinocarveol	1147	1658	-	-	0.22±0.01	0.21±0.01	0.35±0.01	0.76±0.01	0.37±0.01	0.24±0.01	0.66±0.01
Menthone	1148	1462	1.12±0.01	0.24±0.01	0.86±0.01	1.15±0.01	1.54±0.01	1.33±0.01	1.16±0.01	0.94±0.01	0.96±0.01

Camphor	1151	1499	1.23±0.03	0.35±0.01	-	-	-	-	-	-	-
Pinocarvone	1160	1565	-	4.81±0.02	1.74±0.01	2.12±0.01	1.14±0.01	2.31±0.01	1.16±0.01	1.96±0.01	0.96±0.01
Borneol*	1176	1699	-	-	0.37±0.01	2.42±0.01	0.38±0.01	-	0.48±0.01	0.38±0.01	-
Terpinen-4-*ol*	1184	1601	-	-	-	0.22±0.01	-	-	-	-	-
α-Terpineol	1186	1686	0.12±0.01	-	0.57±0.01	0.32±0.01	0.1±0.01	-	-	0.12±0.01	-
Myrtenol	1197	1782	0.31±0.01	-	0.45±0.03	0.31±0.01	-	-	0.28±0.01	0.21±0.01	-
Verbenone	1204	1705	-	1.32±0.02	-	-	-	-	-	-	-
trans-Carveol	1215	1815	-	-	-	0.12±0.02	-	-	-	-	-
β-Cyclocitral	1223	1629	0.33±0.01	-	-	-	-	-	-	-	-
Pulegone	1234	1641	26.23±0.01	13.11±0.01	70.02±0.01	42.90±0.01	51.60±0.01	46.87±0.01	67.25±0.01	63.63±0.01	15.87±0.01
Piperitone	1250	1719	1.23±0.02	2.12±0.01	2.32±0.01	2.17±0.01	3.34±0.01	7.53±0.01	2.21±0.01	3.03±0.01	0.26±0.01
Piperitonene	1340	1882	2.00±0.01	-	-	-	-	-	1.14±0.01	-	-
α-Terpenyl acetate	1349	1685	0.34±0.01	-	-	-	-	-	-	-	-

Piperitenone oxide	1366	1941	33.12±0.01	41.70±0.01	3.08±0.01	13.30±0.01	12.83±0.03	21.68±0.01	5.37±0.01	11.63±0.01	65.21±0.01
**SH**			**3.95**	**10.07**	**1.96**	**10.88**	**6.49**	**2.41**	**1.90**	**5.46**	**1.09**
α-Copaene	1377	1484	0.34±0.01	0.42±0.02	0.26±0.01	0.25±0.01	0.61±0.01	0.95±0.01	0.32±0.01	0.51±0.01	0.25±0.01
β-Bourbonene	1383	1508	-	-	0.41±0.03	0.34±0.05	0.25±0.01	-	0.31±0.01	0.23±0.01	0.22±0.01
β-Elemene	1389	1593	-	1.62±0.01	0.32±0.01	0.93±0.01	1.17±0.01	0.51±0.01	0.24±0.01	0.75±0.01	-
E-Caryophyllene*	1424	1585	0.42±0.01	1.14±0.03	0.35±0.01	1.57±0.01	1.31±0.01	0.42±0.01	0.43±0.01	0.93±0.01	0.43±0.01
β-Copaene	1429	1584	0.51±0.01	0.63±0.01	0.25±0.01	1.13±0.03	-	0.31±0.01	-	0.25±0.01	-
trans-*α*-Bergamotene	1433	1580	-	-	-	-	-	-	-	0.23±0.01	-
(*Z*)-*β*-Farnesene	1454	1639	0.41±0.01	-	-	1.21±0.01	-	-	-	-	-
α-Humulene	1456	1654	-	-	-	-	0.84±0.01	-	-	0.17±0.01	-
allo-Aromadendrene*	1465	1662	-	-	-	1.32±0.01	0.31±0.01	-	-	0.54±0.01	-
Germacrene D*	1481	1692	1.71±0.01	2.61±0.01	0.37±0.01	0.44±0.01	0.52±0.01	0.22±0.01	0.37±0.01	0.32±0.01	0.19±0.01
β-Bisabolene	1494	1729	0.24±0.02	-	-	0.94±0.01	0.54±0.01	-	0.23±0.01	0.51±0.01	-

Viridiflorene	1496	1697	-	-	-	1.14±0.01	-	-	-	-	-
Bicyclogermacrene	1500	1718	-	3.65±0.02	-	-	-	-	-	-	-
β -Curcumene	1514	1731	-	-	-	-	0.94±0.01	-	-	0.71±0.01	-
δ-Cadinene	1517	1745	0.32±0.01	-	-	1.61±0.01	-	-	-	0.31±0.01	-
**OS**			**1.75**	**1.88**	**0.57**	**4.15**	**3.89**	**0.25**	**0.37**	**1.82**	**0.36**
Spathulenol*	1577	2101	-	0.51±0.01	-	0.16±0.01	-	-	-	-	-
Caryophyllene oxide*	1581	1955	1.16±0.01	0.52±0.01	0.31±0.01	1.32±0.01	2.67±0.01	-	-	0.62±0.01	-
γ-Eudesmol	1632	2135	-	0.32±0.03	0.26±0.03	2.16±0.01	0.46±0.01	0.25±0.05	0.37±0.05	0.65±0.01	0.36±0.07
α-Cadinol	1655	2208	0.34±0.01	0.53±0.01	-	0.15±0.05	-	-	-	-	-
α-Bisabolol	1688	2116	0.25±0.03	-	-	0.36±0.01	-	-	-	-	-
α-Bisabolol oxide	1748	2511	-	-	-	-	0.76±0.01	-	-	0.55±0.01	-
**PC**			**0.22**	**0.61**	-	**0.37**	-	**-**	**-**	-	**-**
Carvacrol*	1298	2239	-	-	-	0.37±0.01	-	-	-	-	-

Eugenol*	1370	2175	0.22±0.01	0.61±0.02	-	-	-	-	-	-	-
**CC**			**0.14**	**2.21**	**-**	**0.82**	**0.33**	**-**	**0.32**	**0.22**	**-**
1-Octen-3-*ol*	974	1433	-	2.21±0.01	-	0.61±0.01	0.33±0.01	-	0.32±0.01	0.22±0.01	-
3-Octanol acetate	1125	1376	-	-	-	0.21±0.01	-	-	-	-	-
β-Ionone	1487	1924	0.14±0.01	-	-	-	-	-	-	-	-
**H**			**0.37**	**2.86**	**1.89**	**3.54**	**4.78**	**5.11**	**5.42**	**3.11**	**3.76**
Eicosane*	2000	2000	0.16±0.01	0.22±0.03	0.44±0.01	-	0.35±0.01	0.22±0.01	-	0.24±0.01	3.31±0.01
Heneicosane*	2100	2100	-	0.51±0.01	-	-	-		-	-	-
Docosane*	2200	2200	0.21±0.01	2.13±0.01	1.45±0.03	2.21±0.01	3.81±0.01	4.89±0.03	5.24±0.01	1.72±0.01	3.45±0.01
Tricosane*	2300	2300	-	-	-	0.45±0.01	-	-	-	-	-
Tetracosane*	2400	2400	-	-	-	0.43±0.03	-	-	-	0.38±0.03	-
Pentacosane*	2500	2500	-	-	-	-	0.42±0.01	-	-	0.53±0.01	-
Hexacosane*	2600	2600	-	-	-	0.45±0.01	0.20±0.03	-	0.18±0.01	0.24±0.01	-

Total identified (%)	**91.27**	**90.93**	**92.07**	**95.47**	**91.68**	**93.18**	**92.94**	**97.6**	**92.72**

Retention indices were determined relative to a series of *n*-alkanes (C_8–C_{40}) on capillary columns VF5-ms (RI^a) and CP Wax 52 (RI^b); identification method: RI comparison of RIs with those listed in a homemade library; reported in the literature [87] and/or authentic samples; comparison of mass spectra with those in mass spectral libraries NIST02 [88] and Wiley 9; *,, injection reference compounds; SD, standard deviation; MH, Monoterpene hydrocarbons; OM, Oxygenated monoterpenes; SH, Sesquiterpene hydrocarbons; OS, Oxygenated sesquiterpenes; PC, Phenolic compounds; CC, Carbonylic compounds; H, Hydrocarbons.
