# Supplementary figures and images for: Phytochemicals and Their Correlation with Molecular Data in Micromeria and Clinopodium (Lamiaceae) Taxa
Source: Plants (Basel). 2022 Dec 6;11(23):3407. doi: 10.3390/plants11233407 (PMC9739532; doi:10.3390/plants11233407)

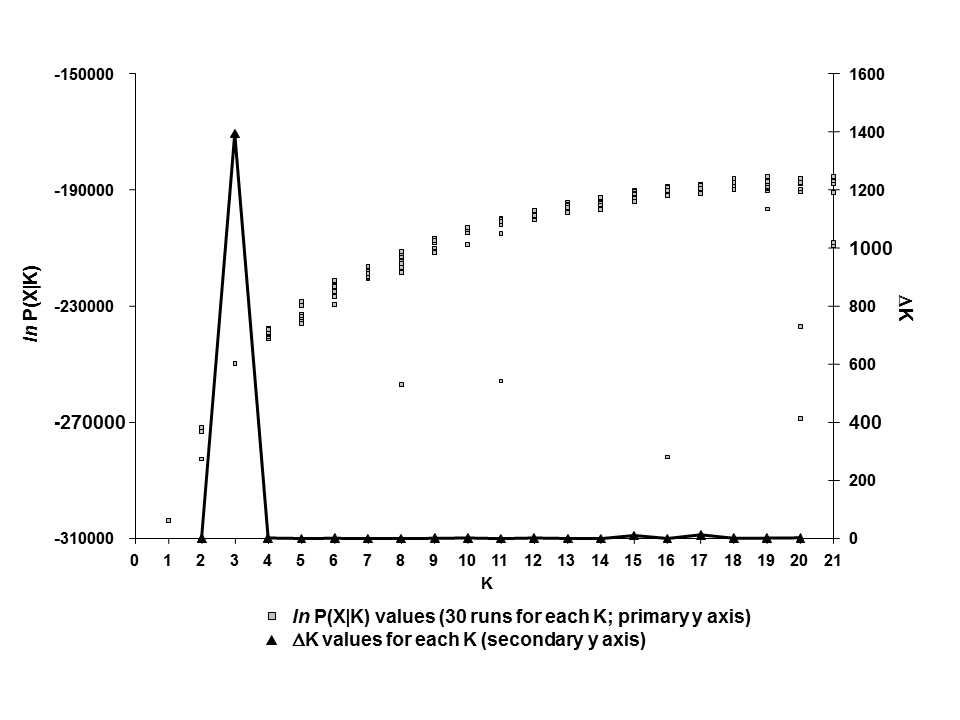

Supplement: Supplementary file 1 [file plants-11-03407-s001.zip › Figure S1.tif]

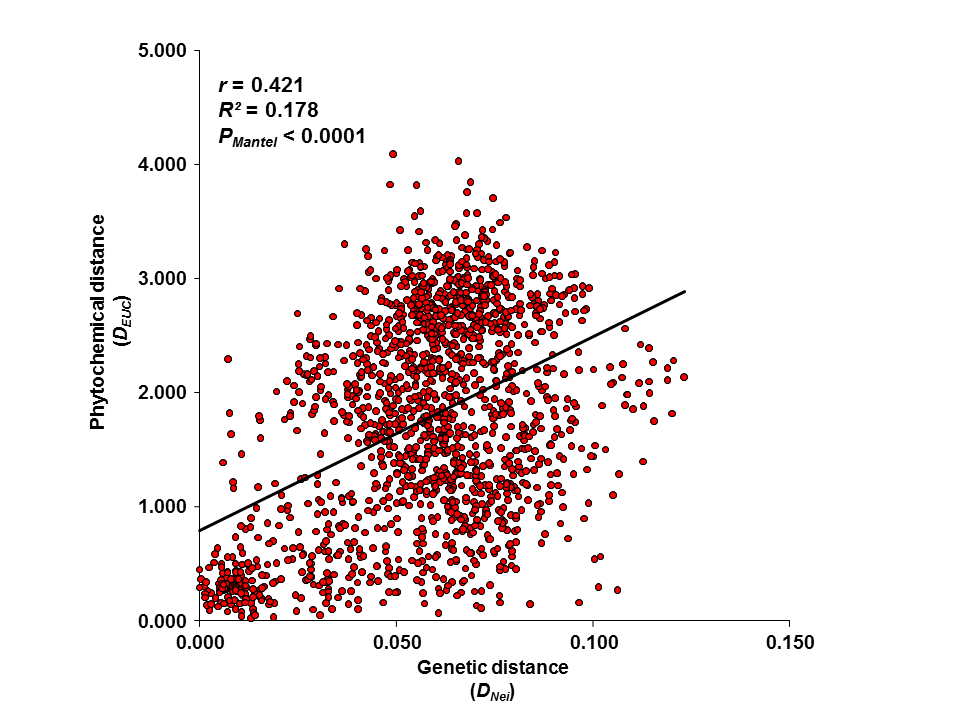

Supplement: Supplementary file 1 [file plants-11-03407-s001.zip › Figure S2.tif]
